# Supplementary material for: Thiolated Hydroxypropyl-β-cyclodextrin: A Potential Multifunctional Excipient for Ocular Drug Delivery
Source: Int J Mol Sci. 2022 Feb 26;23(5):2612. doi: 10.3390/ijms23052612 (PMC8910138; doi:10.3390/ijms23052612)
Supplement: Supplementary file 1 [file ijms-23-02612-s001.zip › ijms-1625473-supplementary.pdf]

Supplementary material

Table S1: Synthesis conditions in which HP-β-CD or thiourea is dissolved in acetic acid or in hydrochloric acid.

Reagents					Reaction Condition				OUTPUT	
HPBCD	AcOH	Thiourea	HCl	AcOH	Vessel	t	time	Power	Yield	Thiolation
mg/mL	Molarity	(mg/ml)	Molarity2	Molarity4	open(O), closed (C)	(°C)	(min)	(W)	%	(umol/g)
100	1.74	133.7	0.4		O	87-80	55	30	24.5	100-150
100	0.174	133.7	0.4		O	85-80	55	30	7.4	80-60
100	8.7	107	0.3	3.5	O	80-75	55	24	5.2	50
100	8.7	107	0.3	3.5	O	75	60	24	2.3	90
100	3.48	133.7	0.4		O	87-77	55	30	10.8	80-100
100	1.74	133.7	0.4		O	85-80	55	30	5.6	160
100	8.7	107	0.3	3.5	O	75	60	30	4.3	115-130
100	0.87	107	0.4		O	85-80	60	30	10.4	90-100
100	0.174	107	0.4		O	85-80	60	30	14	90-100
100	1.74	133.7	0.4		C	85-80	55	30	14.3	100-120
100	0.87	107	0.4		O	80	60	30	9.7	80-90
100	8.7	133.7	1.0	4.3	C	80	60	30	9.8	130-150
100	8.7	133.7	1.0	4.3	C	80-75	55	30	3.1	250-260
100	8.7	133.7	1.0		C	80	45	30	11.9	100-130
100	8.7	133.7	1.0	4.3	C	80	60	40	-	-
100	0.087	133.7	1.0	4.3	C	80	60	40	6	30-50
*100	0.174	200	0.4		O	90	45	40	7.5	120-145
100	0.174	133.7	0.4		O	90	45	40	11.7	100-120
100	8.7	133.7	0.4		C	90	45	40	38	90-115
100	0.174	133.7	1.0	4.3	C	90	45	40	2.8	600
100	0.174	133.7	0.4	0	C	90	45	40	7.1	200-250
100	0.174	133.7	0.4	0	C	90	45	40	5.9	250-260

Table S2: Synthesis conditions in which HP-β-CD or thiourea is dissolved in DMF or in water.

Reagents									Reaction Condition				OUTPUT	
HPBCD	water	DMF	AcOH	Thiourea	water	DMF	HCl	AcOH	Vessel	t	time	Power	Yield	Thiolation
mg/mL	(ml)	ml	Molarity	(mg/ml)	(mL)2	(mL)3	Molarity2	Molarità4	open(O), closed (C)	(°C)	(min)	(W)	%	(umol/g)
100		1		150		3,5	1,5		C	85	45	30	13%	0
100		0,5	8,7	133,7		2,65	1,0	4,3	C	80	45	30	0	-
100	1			133,7	2,45		0,1	6,5	C	80	45	40	0	-
100	1			133,7	2,5			6,5	C	80	45	40	3,4	circa 20
100	1			133,7	3,5			2,2	C	80	45	40	14,5	circa 10
100	1			133,7	3,5			2,2	C	90	45	40	11	20-30
100	1			133,7	2,5			6,5	C	90	45	40	26,1	50-90
100	1			133,7	1,5			10,9	C	90	45	40	36,9	40-60
100	1			133,7	2,45		0,1	6,5	C	90	45	40	4,7	-
100	1			133,7	2,3		0,6	6,5	C	90	45	40	0,5	-
100	1			133,7	2,15		1,0	6,5	C	90	45	40	0	-
100			0,2	200		40			O	90	45	40	237,2	10_20
100			0,2	133,7		40			O	90	45	40	250	10_20
100		1	1,4	150		3,7	0,9		C	90	45	40	27	50-60
100		1	1,4	133,7		3,7	0,9		C	90	45	40	41,1	55-60
100		1		150		3,7	0,9		C	90	45	40	46,1	60-80
100		1		133,7		3,7	0,9		C	90	45	40	37,3	40-50
100			0,2	133,7		3,6	0,4		C	90	45	40	70,1	-
100			0,2	150		4			C	90	45	40	52,5	-
100			0,2	133,7		3,6	0,4		C	90	60	40	54	-
100			0,2	150		4			C	90	60	40	51,4	20-50

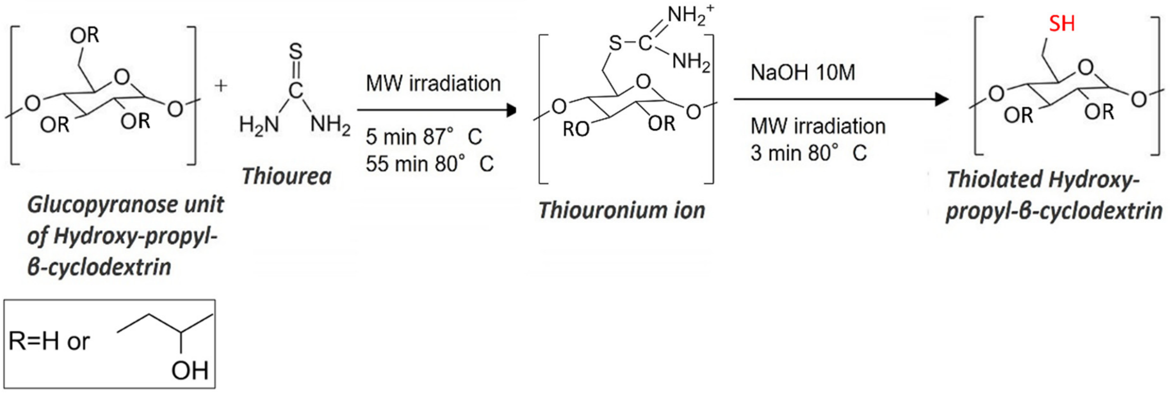

Figure S1 Schematic representation of MW-assisted thiolation. In the first step a solution of thiourea was added dropwise to a solution of HP-β-CD in acidic condition under constant stirring. The obtained solution was irradiated for 5 minutes at 87 °C and 55 minutes at 80 °C. The reaction mixture was than hydrolysed under irradiation for 3 minutes at 80 °C into the thiolated compound.

Figure S1
The scheme should have higher resolution. Moreover, the 'OR' notations are missed at C3 and C6. It should be as in the latter structures.

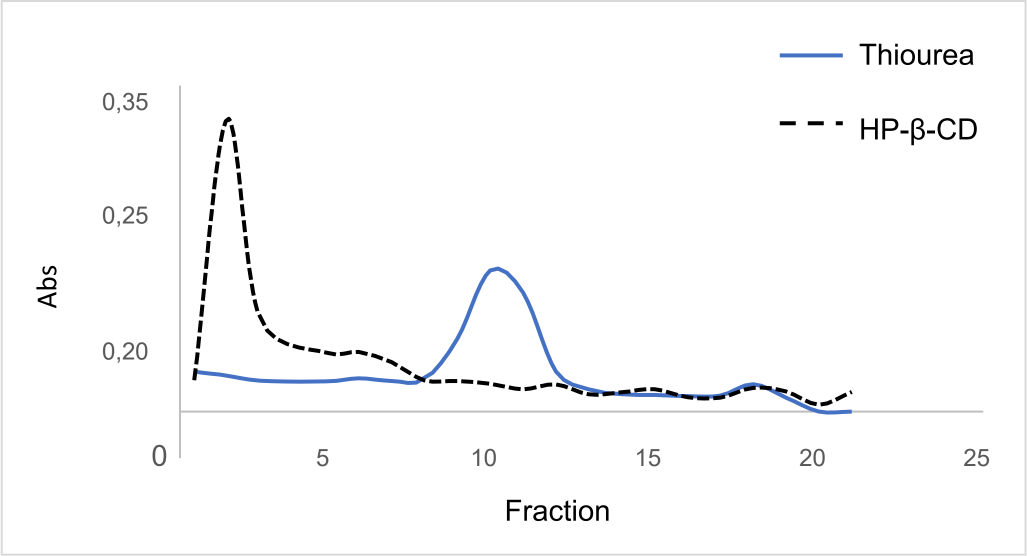

Figure S2. Column elution of thiourea and HPβCD-SH

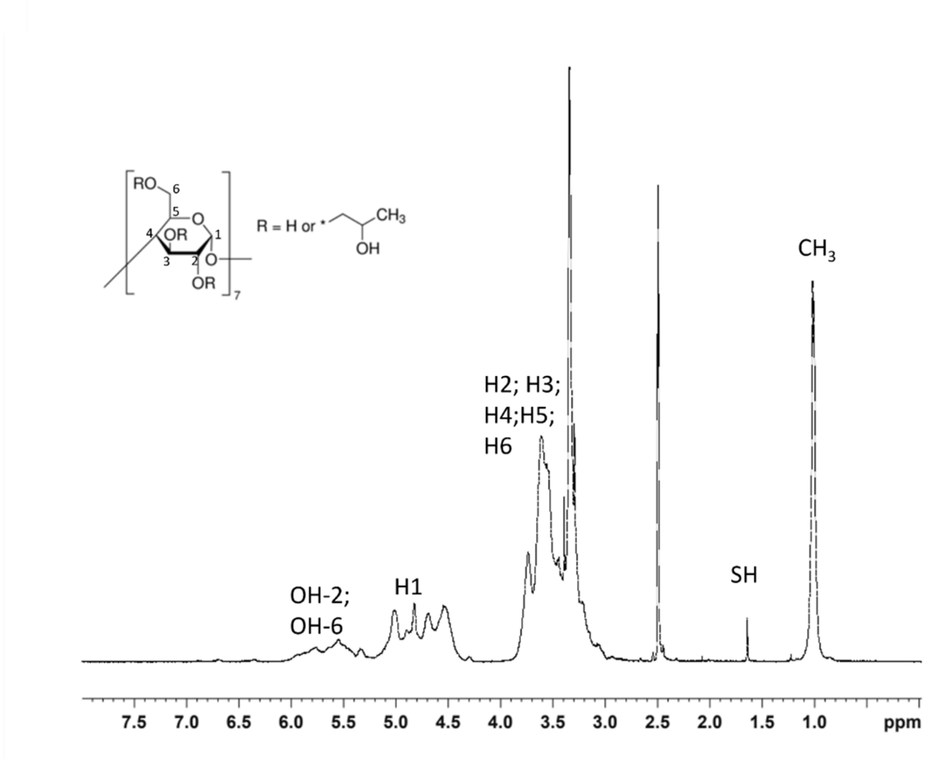

Figure S3. ^{1}H NMR (600 MHz, DMSO-d$_6$, 25 °C, 5 mg/mL) spectra of HP-β-CD-SH. δ = 5.4-6.1ppm (hydroxyl protons in C2 and C6), 5.6-4.9 ppm (anomeric proton), 2.8-3.9 ppm (protons of the pyranosidic ring), 1.65 ppm (proton of the thiol group), 1ppm (methylene protons of the hydroxypropyl moiety).

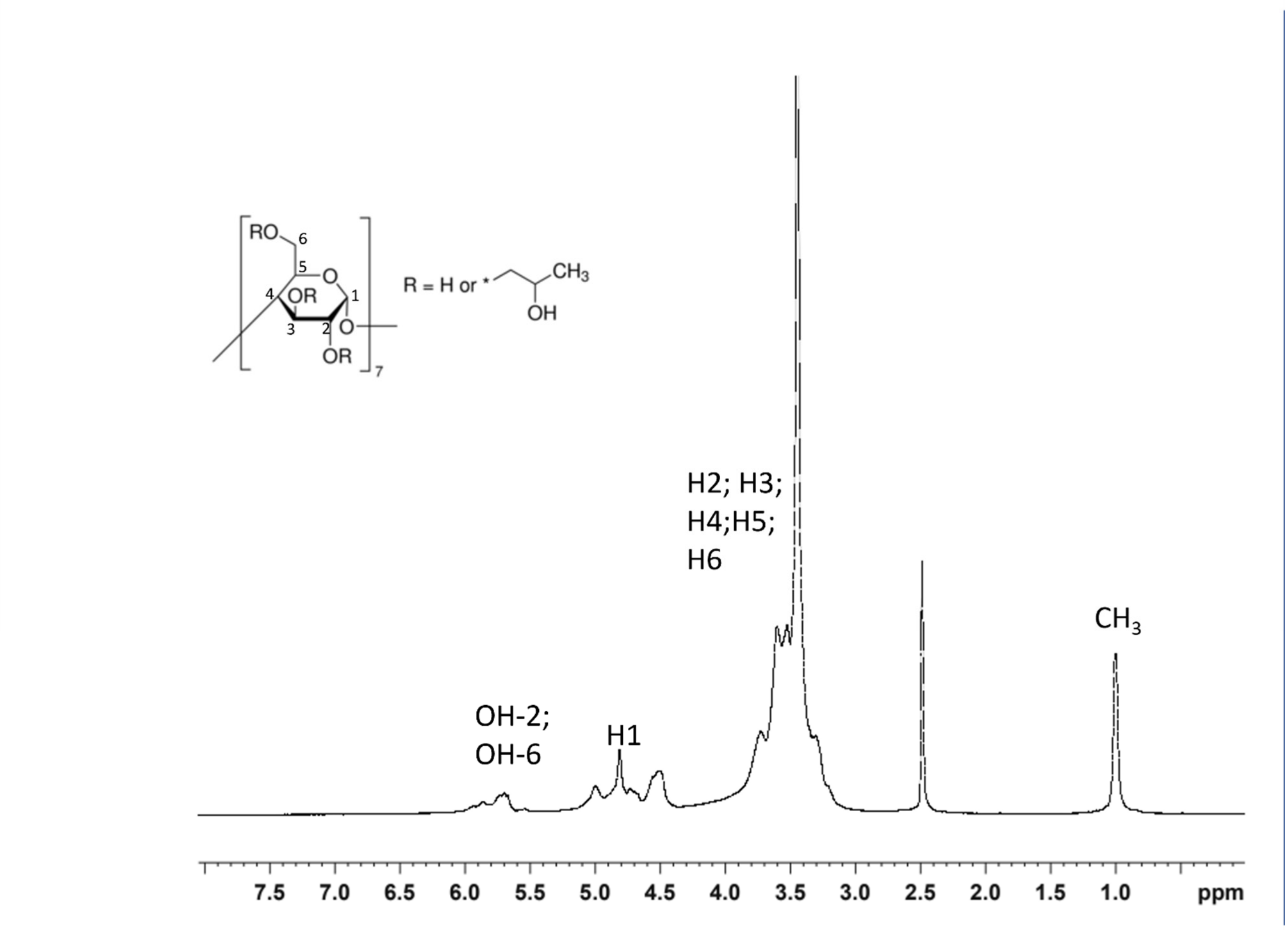

Figure S4. ^{1}H NMR (600 MHz, DMSO-d_6, 25 °C, 5 mg/mL) spectra of HP-β-CD. δ = 5.4-6.1ppm (hydroxyl protons in C2 and C6), 5.6-4.9 ppm (anomeric proton), 2.8-3.9 ppm (protons of the pyranosidic ring), 1ppm (methylene protons of the hydroxypropyl moiety).

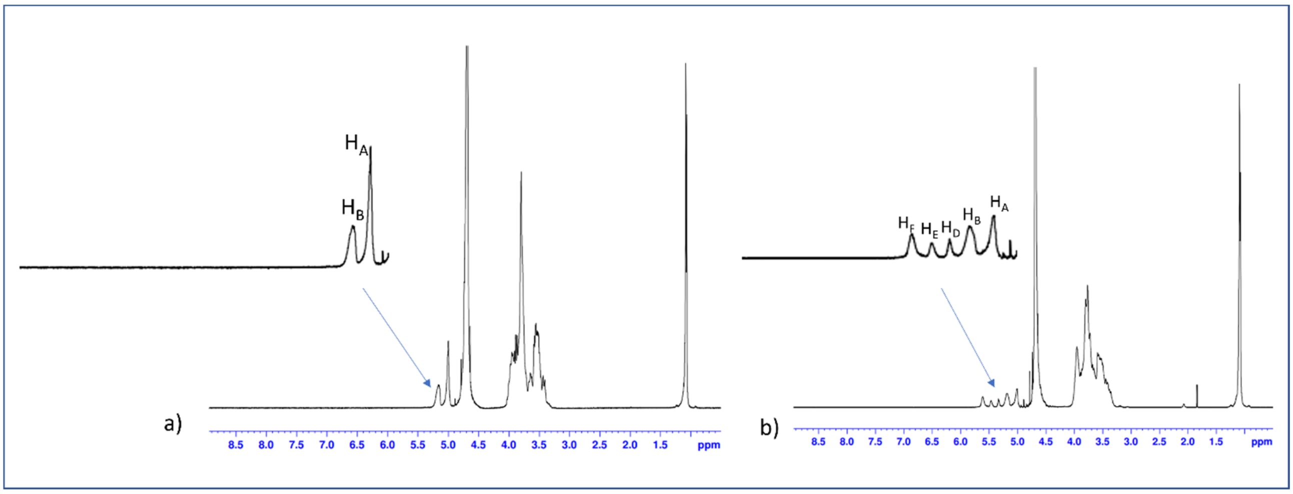

Figure S5. ^{1}H NMR (600 MHz, D_2O, 25 °C, 5 mg/mL) spectra of **(a)** HP-β-CD δ = 5.03ppm, 5.169ppm (anomeric protons of the hydroxypropyl and native β-CD), 2.8-3.9 ppm (protons of the pyranosidic ring), 1ppm (methylene protons of the hydroxypropyl moiety). **(b)** HP-β-CD-SH δ = 5.03ppm, 5.169ppm (anomeric protons of the hydroxypropyl and native β-CD), 5.34ppm, 5.45ppm, 5.61ppm 2.8-3.9 ppm (anomeric protons of thiolated cyclodextrin, with or without the hydroxypropyl functionality, substituted in C6 or C2), 2.8-4.1 ppm (protons of the pyranosidic ring), 1.65ppm (proton of the thiol group), 1ppm (methylene protons of the hydroxypropyl moiety).
